# *Klebsiella pneumoniae* from community-acquired infections and an urban river share antimicrobial resistance profiles and high-risk lineages but differ in virulence

**DOI:** 10.1007/s00203-026-05239-0

**Published:** 2026-10-06

**Authors:** Adriano Souza Santos Monteiro, João Ricardo Filho, Vívian Santos Galvão, Vanessa Tibolla Moretto, Ana Beatriz Purificação, Lee Senhorinha Almeida Andrade, Camila Maria Piñeiro Silva, Lúcio Macedo Barbosa, Luciano Kalabric Silva, Soraia Machado Cordeiro, Ronald Edward Blanton, Joice Neves Reis, Mitermayer Galvão Reis

**Affiliations:** 1 Laboratory of Pathology and Molecular Biology, Gonçalo Moniz Institute, Oswaldo Cruz Foundation, Salvador, Bahia Brazil; 2 Department of Biointeraction, Institute of Health Sciences, Federal University of Bahia, Salvador, Bahia Brazil; 3 Postgraduate Program in Human Pathology, Gonçalo Moniz Institute, Oswaldo Cruz Foundation, Salvador, Bahia Brazil; 4 Faculty of Pharmacy, Federal University of Bahia, Salvador, Bahia Brazil; 5 Postgraduate Course in Biotechnology in Health and Investigative Medicine, Gonçalo Moniz Institute, Oswaldo Cruz Foundation, Salvador, Bahia Brazil; 6 Bahiana School of Medicine and Public Health, Salvador, Bahia Brazil; 7 Postgraduate Program in Microbiology, Institute of Biology, Federal University of Bahia, Salvador, Bahia Brazil; 8 Department of Tropical Medicine, Tulane School of Public Health and Tropical Medicine, New Orleans, LA USA; 9 Faculty of Medicine, Federal University of Bahia, Salvador, Bahia Brazil; 10 Yale School of Public Health, New Haven, Connecticut USA

**Keywords:** Multidrug resistance, Environmental reservoirs, Hypervirulence, Resistance genes, β-lactamase, Multilocus sequence typing

## Abstract

*Klebsiella pneumoniae* poses a significant public health threat, but the relationship between community-acquired (CA) infections and environmental isolates remains poorly understood. Therefore, this study aimed to assess whether *K. pneumoniae* from CA infections share microbiological and molecular features with isolates recovered from an urban river in Salvador, Brazil. A total of 115 isolates, including 65 from CA infections and 50 from river water, were analyzed using antimicrobial susceptibility testing, β-lactamase and virulence gene detection, and multilocus sequence typing (MLST). Resistance rates were similar between groups, including multidrug resistance (24.6% vs. 22.0%; *p* > 0.05) and carbapenem resistance (4.6% vs. 4.0%; *p* > 0.05). β-lactamase gene detection was consistent with observed resistance patterns, with *bla*_CTX−M_ detected in 18.5% of CA isolates and 12.0% of river isolates, while *bla*_KPC−2_ (3.1% vs. 2.0%) and *bla*_NDM−1_ (1.5% vs. 2.0%) occurred at comparable frequencies. However, hypervirulent *K. pneumoniae* isolates were identified only among CA infections (4.6%). MLST revealed high clonal diversity, with 88 sequence types (STs), including 14 novel STs, with most STs being singletons. Four STs were shared between groups, including the high-risk lineages ST11 and ST307. These findings indicate that clinically relevant *K. pneumoniae* lineages, including high-risk, occur beyond healthcare settings, with urban aquatic environments serving as potential environmental reservoirs.

**Supplementary Information:**

The online version contains supplementary material available at https://doi.org/10.1007/s00203-026-05239-0.

## Introduction

*Klebsiella pneumoniae* is a major global pathogen and a leading cause of morbidity and mortality worldwide, largely driven by its high rates of antimicrobial resistance. Notably, carbapenem-resistant Enterobacterales, including *K. pneumoniae*, are classified as critical-priority pathogens in the World Health Organization (WHO) 2024 Bacterial Priority Pathogens List (World Health Organization 2024). This public health impact is primarily associated with the widespread dissemination of extended-spectrum β-lactamases (ESBLs) and carbapenemases, which severely limit therapeutic options and facilitate the global spread of multidrug-resistant (MDR) isolates. In parallel, the emergence of hypervirulent *K. pneumoniae* (hv*Kp*) lineages has further expanded its clinical and public health relevance, with increasing evidence of the convergence between hypervirulence and antimicrobial resistance (Zhang et al. 2020; Monteiro et al. 2024).

*K. pneumoniae* is also a common colonizer of the human gastrointestinal tract and can consequently be detected in human feces, with fecal carriage rates in healthy populations reported at 5–35% in Western community settings and up to 88% in some Asian settings, depending on geographic region, detection method, and population studied (Russo and Marr 2019; Bray and Zafar 2024). Intestinal carriage is epidemiologically relevant, as colonizing strains may serve as a source of subsequent infection (Bray and Zafar 2024) and may enter the environment through fecal shedding.

Consistent with this route of dissemination, antimicrobial-resistant *K. pneumoniae* has increasingly been detected in environmental compartments, particularly in urban rivers impacted by fecal contamination and untreated effluents (Moretto et al. 2021). Concurrently, a limited number of high-risk lineages, internationally associated with multidrug resistance and hospital outbreaks, have disseminated globally and contribute substantially to the clinical burden of *K. pneumoniae* (Wyres et al. 2020). Of particular concern is the increasing convergence of successful MDR lineages with hypervirulence-associated determinants. Determining whether these high-risk lineages and virulence determinants historically associated with hv*Kp* also circulate beyond clinical environments is therefore relevant not only for antimicrobial resistance surveillance but also for understanding their distribution across ecological niches. From a One Health perspective, assessing whether resistance profiles, virulence determinants, and high-risk lineages detected in environmental settings are also found among community-derived isolates is particularly important, as comparative studies have primarily relied on hospital-derived isolates, often pre-selected for antimicrobial resistance (Runcharoen et al. 2017; Khan et al. 2018; Lepuschitz et al. 2019; Zou et al. 2023) and therefore strongly influenced by hospital-specific selective pressures. Comparative data addressing community and environmental reservoirs remain particularly scarce in Brazil.

This study aimed to assess whether *K. pneumoniae* isolates responsible for community-acquired (CA) infections share microbiological and molecular characteristics with isolates recovered from an urban river in Salvador, Brazil.

## Materials and methods

### Study design and isolate sources

This is a cross-sectional study comparing environmental samples from an urban river with clinical isolates obtained from CA infections. Clinical isolates were obtained from patients with CA bloodstream infections (CA-BSI) between March 2015 and May 2019 and CA urinary tract infections (CA-UTI) between April 2019 and July 2023 in Salvador, Brazil. CA-BSI isolates were obtained from a previous cross-sectional study conducted in two tertiary hospitals in Salvador, whereas CA-UTI isolates were obtained from a surveillance study conducted at the Clinical Analysis Laboratory of the School of Pharmacy, Federal University of Bahia. All participants were from Salvador, the same city where the Cobre River watershed is located. However, clinical isolates were not selected based on patient residence or proximity to the Cobre River, and no epidemiological link between patients and environmental sampling sites was assessed. CA infections were defined according to the criteria proposed by Friedman et al. (2002), as infections occurring in patients without previous healthcare exposure. BSI were diagnosed based on positive blood cultures within 48 h after hospitalization. Blood cultures were processed using the BacT/Alert^®^ system (bioMérieux), and UTI were defined by urine cultures yielding ≥ 100,000 CFU/mL (colony-forming units/mL). Exclusion criteria included prior intravenous therapy, renal dialysis, wound care, or specialized nursing care within 30 days before infection; surgery or hospitalization within the previous 90 days; residence in nursing homes or long-term care facilities within the past 12 months; and invasive urinary procedures or long-term urinary catheter use within 30 days before infection.

**Fig. 1:**
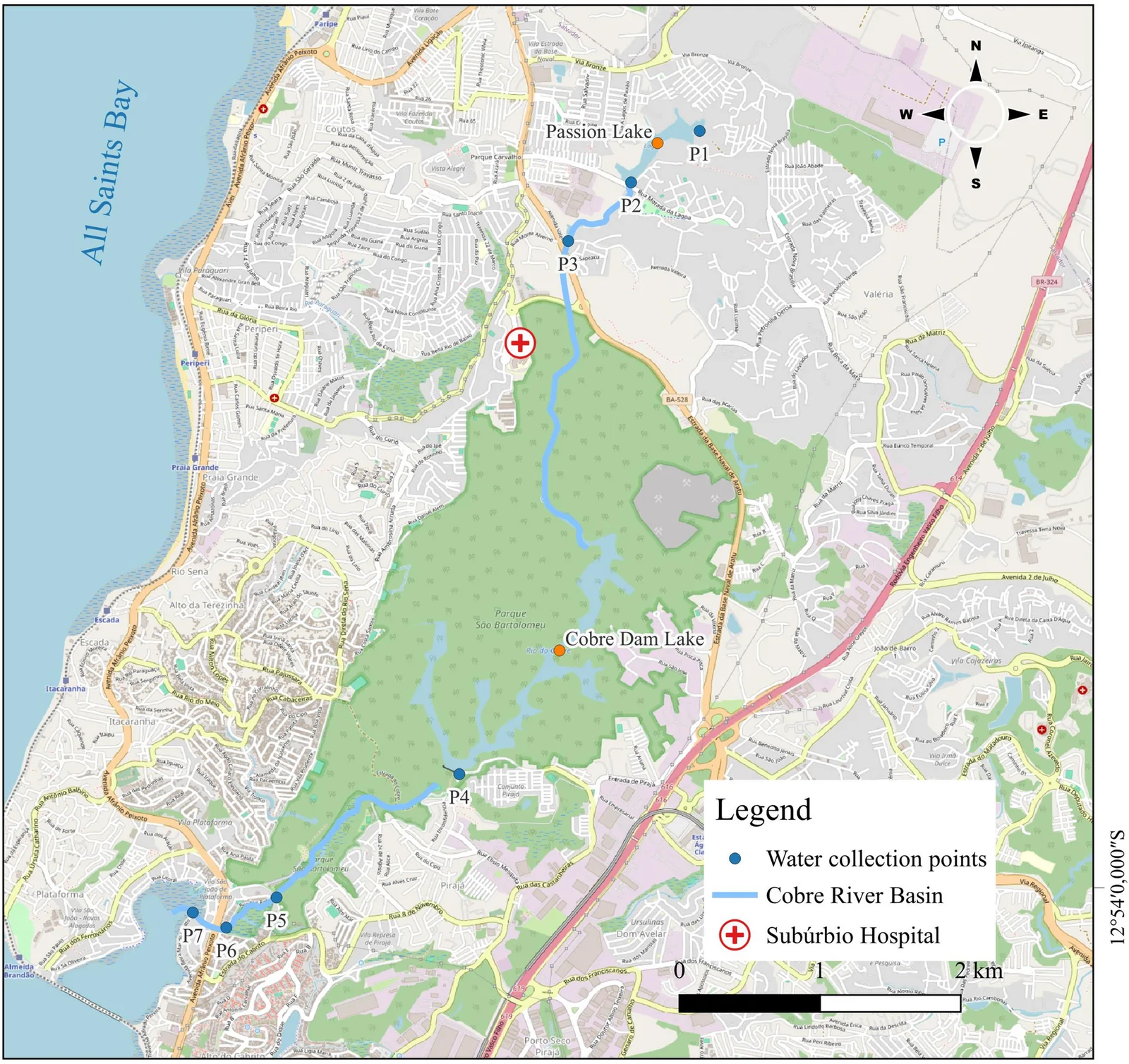
Spatial distribution of environmental sampling sites along the Cobre River watershed, Salvador, Brazil. Map created by the authors using QGIS software. Base map and data from OpenStreetMap and OpenStreetMap Foundation are available under the Open Database License (ODbL). Cartography is licensed under the Creative Commons Attribution-ShareAlike 2.0 license (CC BY-SA 2.0). Geographic outlines and base data are available at: https://www.openstreetmap.org. Attribution: OpenStreetMap contributors

Environmental isolates were obtained from water samples collected from the Cobre River watershed, an open urban water system located in Salvador, Brazil. The Cobre River is affected by anthropogenic inputs, and localized direct discharges of untreated domestic wastewater were observed in some areas along its course. Sampling was performed at seven sites along the river, including the spring (river source), five downstream sites, and the river mouth (Fig. 1). Four sampling campaigns were conducted at approximately three-month intervals on October 27, 2021, January 24, 2022, May 5, 2022, and August 5, 2022. During each campaign, one water sample was collected at each of the seven sampling sites, yielding a total of 28 environmental water samples. Samples were collected between 8:00 a.m. and 1:00 p.m., transported on ice, and processed within 2 h of collection for coliform enumeration and bacterial culture. Coliforms were quantified using a chromogenic enzyme-based medium (Coliscan Easygel, Micrology Laboratories, USA). One milliliter of each water sample was added to the liquid Coliscan Easygel medium provided with the kit and gently homogenized by inversion. The entire sample-medium mixture was then poured into a sterile Petri dish and allowed to solidify according to the manufacturer’s instructions. Plates were incubated at 36 ± 2 °C for 24 h, after which colonies were manually counted, and the results were expressed as CFU/mL. *Escherichia coli* was differentiated from other coliforms based on colony color, according to the manufacturer’s interpretation criteria.

For bacterial isolation, serial dilutions of water samples were plated independently on MacConkey agar supplemented with 2 µg/mL cefotaxime and on CHROMagar™ Orientation without antibiotic. Of the 50 environmental isolates, 9 were recovered from cefotaxime-supplemented MacConkey agar and 41 from CHROMagar™ Orientation without antibiotic. Plates were incubated at 36 ± 2 °C for 24 h. All colonies with morphology and color characteristics compatible with *K. pneumoniae* recovered from each water sample were selected for further identification. Isolates with identical phenotypic and genotypic profiles, including antimicrobial resistance profiles, virulence determinants, and multilocus sequence typing (MLST), were considered duplicate isolates for the purposes of this analysis, and only one representative was retained in the final dataset.

### Bacterial identification and antimicrobial susceptibility testing

Clinical isolates were identified by MALDI-TOF MS, the VITEK^®^ system, or standard biochemical tests. Antimicrobial susceptibility testing was performed using the VITEK^®^ system or the disk diffusion method, and results were interpreted according to the Clinical and Laboratory Standards Institute (CLSI) guidelines (CLSI 2023). Environmental isolates recovered from Cobre River water were identified by MALDI-TOF MS, and antimicrobial susceptibility testing was performed using the VITEK^®^ 2 system. Although environmental isolates were originally interpreted according to BrCAST criteria (https://brcast.org.br), the available MIC values were subsequently reinterpreted using CLSI breakpoints to ensure consistency with the clinical isolates. This reinterpretation did not result in changes in resistance categorization; therefore, all antimicrobial susceptibility results reported in this study were interpreted according to CLSI criteria. ESBL production was confirmed using the CLSI phenotypic confirmatory disc test. Multidrug resistance was defined for both clinical and environmental isolates as acquired non-susceptibility to at least one agent in three or more antimicrobial categories, according to Magiorakos et al. (2012).

### Detection of β-lactamase and virulence genes

DNA extracted from bacterial isolates using the boiling method (Dallenne et al. 2010) was used as the standard template for all conventional PCR assays. For a limited number of isolates subjected to MLST analysis, purified DNA was additionally obtained using the QIAamp DNA Mini Kit (Qiagen) for downstream amplification and sequencing. Major β-lactamase genes of clinical and epidemiological relevance in *K. pneumoniae*, including ESBL- and carbapenemase-associated genes, were investigated by conventional PCR targeting *bla*_TEM_, *bla*_SHV_, *bla*_OXA−1−like_, *bla*_CTX−M_ groups 1, 2, and 9, *bla*_GES_, *bla*_OXA−48_, *bla*_IMP_, *bla*_VIM_, *bla*_KPC_, and *bla*_NDM_ (Dallenne et al. 2010; Gondal et al. 2020; Carattoli et al. 2021). *bla*_KPC_ and *bla*_NDM_ amplicons were further characterized by Sanger sequencing to determine allele variants.

Virulence-associated genes were screened by conventional PCR, including genes associated with capsular serotypes K1/K2 (*magA* and *wzi_K2*), capsule regulation and biosynthesis (*rmpA*, *rmpA2*, *wabG*, and *uge*), siderophore systems (*entB*, *ybtS*, *iucA*, and *iroB*), fimbriae (*fimH* and *mrkD*), iron uptake (*kfu*), allantoin metabolism (*allS*), urease production (*ureA*), putative metabolite transport (*peg-344*), serum resistance (*traT*), and toxin production (*hlyA* and *cnf-1*) (Brisse et al. 2009; El Fertas-Aissani et al. 2013; Compain et al. 2014; Russo et al. 2018). Isolates positive for *peg-344*, *iroB*, *iucA*, *rmpA*, and *rmpA2* were classified as hv*Kp*, whereas the remaining isolates were classified as classical *K. pneumoniae* (c*Kp*) (Russo et al. 2024). Previously characterized positive-control isolates and a no-template control were included in all PCR assays. Primer sequences and conditions for all PCR reactions are provided in Table S1 (Online Resource 1).

### Molecular typing

MLST was performed by sequencing the housekeeping genes *gapA*, *infB*, *mdh*, *pgi*, *phoE*, *rpoB*, and *tonB*, according to the *K. pneumoniae* MLST scheme available at the Institut Pasteur database (https://bigsdb.pasteur.fr/klebsiella/) (Diancourt et al. 2005). Relationships among sequence types (STs) were visualized by constructing a minimum spanning tree using the goeBURST algorithm implemented in PHYLOViZ 2.0 (Nascimento et al. 2017). Novel STs were submitted to the Institut Pasteur MLST database for curation and assignment.

### Statistical analysis

Descriptive analyses were performed using frequencies and measures of central tendency (median) and dispersion (interquartile range [IQR]). Statistical analyses were conducted using the chi-square test or Fisher’s exact test, as appropriate. A p-value < 0.05 was considered statistically significant, and all analyses were conducted using GraphPad Prism version 8.0.1 (GraphPad Software, USA).

## Results

A total of 115 *K. pneumoniae* isolates were included in this study, comprising 65 from CA infections and 50 from river water samples collected in Salvador, Brazil. Among the clinical isolates, 41 originated from CA-UTI and 24 from CA-BSI. These infections occurred in adult patients, with a median age of 47 years (first quartile 24, third quartile 66) for CA-UTI and 76 years (first quartile 62, third quartile 83) for CA-BSI. Females predominated in the CA-UTI group (36/41; 87.8%), whereas males were more frequent in the CA-BSI group (16/24; 66.7%).

Environmental isolates were recovered from multiple sampling sites along the Cobre River. The spatial distribution of *K. pneumoniae* followed the downstream pattern observed for fecal contamination indicators, with more isolates recovered at sites presenting higher levels of total coliforms and *E. coli* (Table 1).

**Table 1:** Fecal contamination indicators and recovery of *Klebsiella pneumoniae* along the urban river sampling sites, Salvador, Brazil

Sampling site	Total coliforms (CFU/mL, median (IQR))	*Escherichia coli* (CFU/mL, median (IQR))	*K. pneumoniae* isolates recovered (*n*)*
Site 1	390.0 (272.5–442.5)	1.5 (0.0–4.3)	1
Site 2	105.5 (85.8–115.0)	1.0 (1.0–1.0)	2
Site 3	92.5 (48.8–142.5)	12.5 (4.0–20.0)	2
Site 4	25.0 (17.0–33.8)	2.5 (0.75–5.5)	1
Site 5	724.5 (695.0–1041.8)	380.0 (278.75–537.5)	14
Site 6	1026.0 (917.5–1190.8)	973.5 (917.5–1010.8)	10
Site 7	1210.0 (960.0–1448.5)	1057.0 (885.0–1190.5)	20

*IQR* interquartile range

*K. pneumoniae isolates recovered represent the final number of isolates included after exclusion of clonal isolates based on phenotypic and genotypic profiles

All isolates underwent phenotypic antimicrobial susceptibility testing, and resistance rates (Table 2) and individual phenotypic resistance profiles (Table S2, Online Resource 1) were compared. Overall, resistance rates were broadly similar between CA infection and environmental isolates across the antimicrobial agents tested, although these comparisons should be interpreted cautiously given the use of cefotaxime-supplemented medium for the recovery of some environmental isolates. The proportion of MDR isolates was 24.6% (16/65) among CA infection isolates and 22.0% (11/50) among river water isolates. Notably, carbapenem-resistant isolates were identified in both sources, with resistance detected in 4.6% of CA infection isolates and 4.0% of environmental isolates. Among environmental isolates, individual phenotypic resistance profiles varied across sampling sites without evidence of a consistent site-specific pattern. Resistant and MDR isolates were recovered from several sampling sites, including sites with elevated total coliform and *E. coli* counts (Table S3, Online Resource 1).

**Table 2:** Antimicrobial susceptibility profiles of *Klebsiella pneumoniae* isolates from community-acquired infections (*n* = 65) and river water (*n* = 50)

Antimicrobial agent	Community-acquired infections	River water	*p*-value
	Resistance (%)	Resistance (%)	
	*n* = 65	*n* = 50	
Amoxicillin-clavulanic acid	11 (16.9)	11 (22.0)	0.63
Piperacillin-tazobactam	9 (13.8)	6 (12.0)	1.00
Ceftriaxone	13 (20.0)	10 (20.0)	1.00
Ceftazidime	13 (20.0)	9 (18.0)	0.82
Cefepime	14 (21.5)	8 (16.0)	0.49
Aztreonam	9 (13.8)	10 (20.0)	0.45
Ciprofloxacin	16 (24.6)	10 (20.0)	0.66
Gentamicin	7 (10.8)	4 (8.0)	0.75
Amikacin	1 (1.5)	0 (0.0)	1.00
Imipenem	3 (4.6)	2 (4.0)	1.00
Meropenem	3 (4.6)	2 (4.0)	1.00
Multidrug-resistant	16 (24.6)	11 (22.0)	0.83

P-values were calculated using Pearson’s chi-square test or Fisher’s exact test, as appropriate

**Fig. 2:**
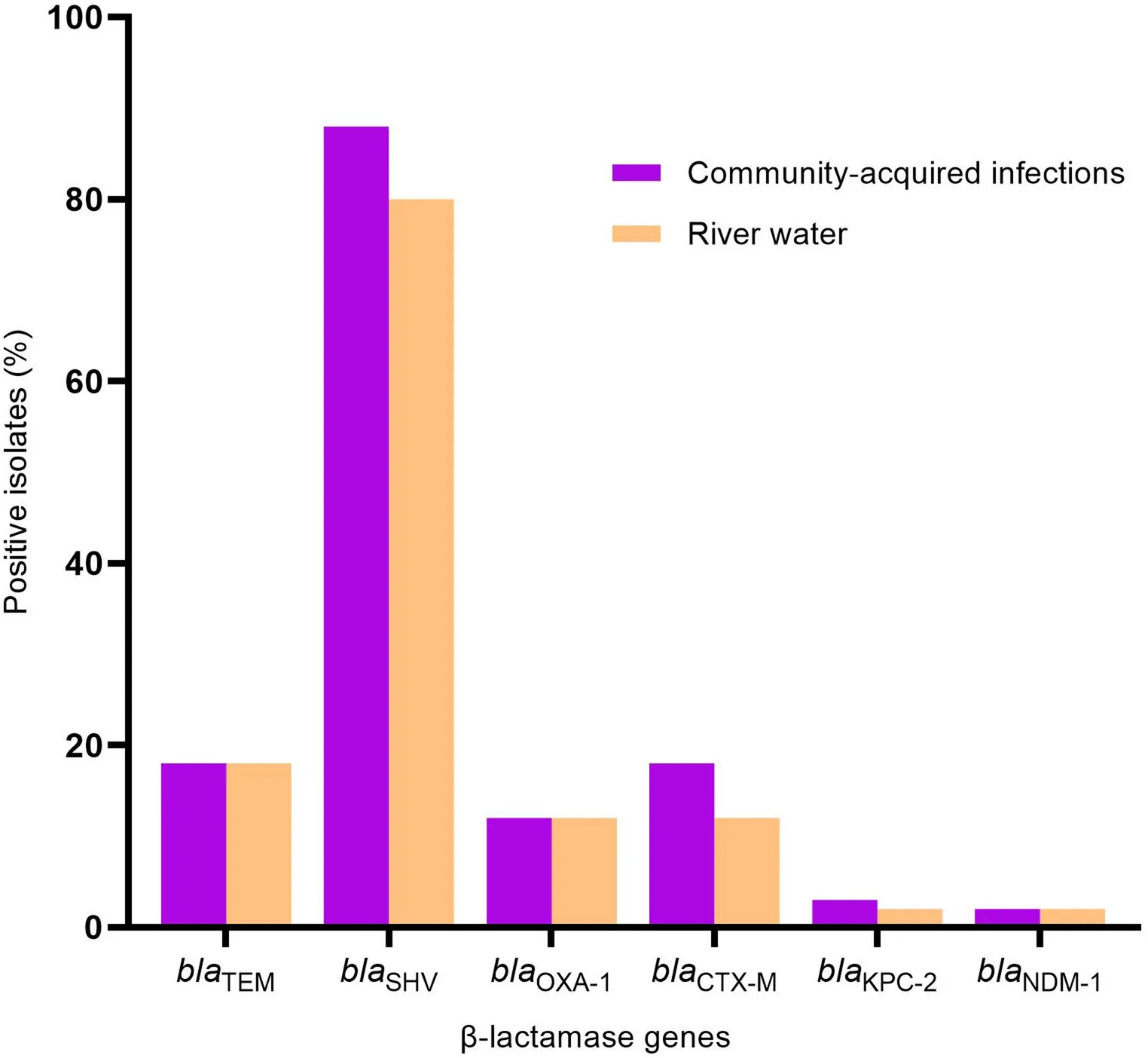
Comparative distribution of β-lactamase genes among *Klebsiella pneumoniae* isolates obtained from community-acquired infections (*n* = 65) and river water (*n* = 50)

The distribution of β-lactamase genes was consistent with the observed phenotypic resistance patterns (Fig. 2). *bla*_CTX−M_ genes were detected in 18.5% (12/65) of CA infection isolates and 12.0% (6/50) of river water isolates. CTX-M group 1 was the most frequently detected, occurring in 15.4% (10/65) and 12.0% (6/50) of isolates, respectively, whereas CTX-M group 9 was detected exclusively in two CA infection isolates. All phenotypically identified ESBL-producing isolates carried a *bla*_CTX−M_ gene. Carbapenemase genes were detected in isolates from both sources. *bla*_KPC−2_ was identified in two CA infection isolates and one river isolate, whereas *bla*_NDM−1_ was detected in one isolate from each source.

Other β-lactamase genes, including *bla*_TEM_ (12/65; 18.5% vs. 9/50; 18.0%), *bla*_SHV_ (57/65; 87.7% vs. 40/50; 80.0%), and *bla*_OXA−1−like_ (8/65; 12.3% vs. 6/50; 12.0%), were more frequently detected among CA infection isolates, although without significant variation between groups. Among river water isolates, β-lactamase genes showed a heterogeneous distribution across sampling sites, with carbapenemase genes restricted to Site 7 (Table S4, Online Resource 1).

In contrast to the similar distribution of resistance-associated phenotypes and determinants, the distribution of virulence-associated genes showed statistically significant differences in prevalence. Hv*Kp* isolates were identified exclusively among those from CA infections (4.6%; 3/65), although without statistical significance. Notably, all hv*Kp* isolates were susceptible to all antimicrobial agents tested. Among the virulence markers, *ybtS* (yersiniabactin siderophore) was significantly more frequent in CA isolates (29.2%; 19/65) than in river isolates (8.0%; 4/50; *p* < 0.01) and *entB* (enterobactin siderophore) was also more prevalent in CA isolates (98.5%; 64/65) than in river isolates (88.0%; 44/50; *p* = 0.04). The remaining virulence genes were detected at comparable frequencies in both groups (Table 3).

**Table 3:** Distribution of virulence-associated genes among *Klebsiella pneumoniae* isolates from community-acquired infections (*n* = 65) and river water (*n* = 50)

Related virulence factor	Target genes	Community-acquired infections	River water	*p*-value
		*n* = 65 (%)	*n* = 50 (%)	
Capsular serotype K1	*magA*	1 (1.5)	0 (0.0)	1.00
Capsular serotype K2	*wzi*_K2	3 (4.6)	0 (0.0)	0.26
Capsule	*rmpA*	3 (4.6)	0 (0.0)	0.26
Capsule	*rmpA2*	2 (3.1)	0 (0.0)	0.50
Lipopolysaccharide	*wabG*	65 (100)	48 (96.0)	0.19
Lipopolysaccharide	*uge*	59 (90.8)	43 (86.0)	0.56
Enterobactin	*entB*	64 (98.5)	44 (88.0)	**0.04**
Yersiniabactin	*ybtS*	19 (29.2)	4 (8.0)	**< 0.01**
Aerobactin	*iucA*	3 (4.6)	0 (0.0)	0.26
Salmochelin	*iroB*	3 (4.6)	0 (0.0)	0.26
Type 1 fimbria	*fimH*	65 (100)	47 (94.0)	0.08
Type 3 fimbria	*mrkD*	61 (93.8)	46 (92.0)	0.73
Iron uptake system	*kfu*	20 (30.8)	19 (38.0)	0.43
Allantoin metabolism	*allS*	3 (4.6)	2 (4.0)	1.00
Urease	*ureA*	65 (100)	49 (98.0)	0.44
Putative metabolite transporter	*peg-344*	3 (4.6)	0 (0.0)	0.26
Serum resistance	*traT*	-	-	-
Alpha hemolysin	*hlyA*	-	-	-
Cytotoxic necrotizing factor-1	*cnf-1*	-	-	-
c*Kp*	*	62 (95.4)	50 (100)	0.26
hv*Kp*	*	3 (4.6)	0 (0.0)	

*Isolates positive for the *peg-344*, *iroB*, *iucA*, *rmpA*, and *rmpA2* genes were considered hv*Kp*. The remaining isolates were considered c*Kp*

P-values were calculated using Pearson’s chi-square test or Fisher’s exact test, as appropriate. Bold values indicate statistically significant differences (p < 0.05).

**Fig. 3:**
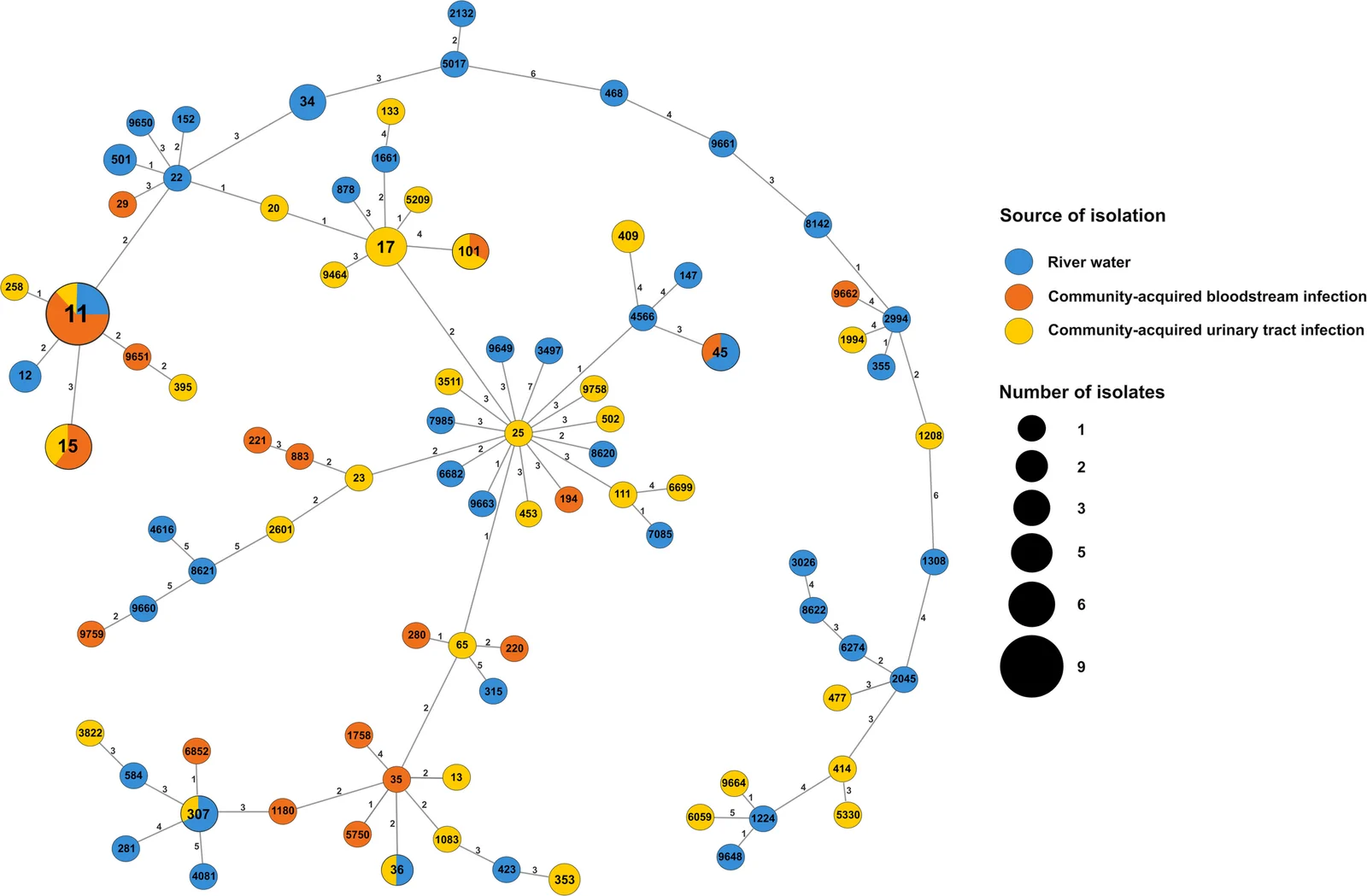
goeBURST minimum spanning tree based on MLST profiles showing the genetic relationships among 115 *K. pneumoniae* isolates identified in this study, comprising 88 STs. Each circle represents a distinct ST, with circle size proportional to the number of isolates. Colors indicate the source of isolates: blue represents river water, orange represents community-acquired bloodstream infections, and yellow represents community-acquired urinary tract infections. Numbers on the connecting lines represent allelic differences between linked STs. Mixed colors indicate STs detected in more than one source

A total of 88 distinct STs were identified among 115 isolates, indicating a high level of genetic diversity within the studied population. The most frequent STs were ST11 (*n* = 9), ST15 (*n* = 6), and ST17 (*n* = 5), followed by ST34, ST45, ST101, and ST307 (*n* = 3 each), whereas most STs were represented by single isolates. Additionally, 14 novel STs were identified in this study, namely ST8620, ST8621, ST8622, ST9648, ST9649, ST9650, ST9651, ST9660, ST9661, ST9662, ST9663, ST9664, ST9758, and ST9759 (Table S5, Online Resource 1). Four STs were shared between river water and CA infection isolates: ST11, ST36, ST45, and ST307 (Fig. 3). Among the shared STs, ST11 and ST307, both globally disseminated high-risk lineages, included isolates harboring ESBL- and carbapenemase-associated genes: *bla*_CTX−M_ group 1, *bla*_KPC−2_, and *bla*_NDM−1_ among ST11 isolates, and *bla*_CTX−M_ group 1 among ST307 isolates from both settings (Table 4).

**Table 4:** Distribution of ESBL- and carbapenemase-associated genes among shared sequence types of *Klebsiella pneumoniae* from community-acquired infections and river water

ID	ST	Source	ESBL-associated genes	Carbapenemase-associated genes
ENV34	ST11	River water	-	*bla*_NDM−1_
ENV46	ST11	River water	*bla*_CTX−M_ group 1	-
CAB03	ST11	Community-acquired infections	*bla*_CTX−M_ group 1	-
CAB12	ST11	Community-acquired infections	-	-
CAB14	ST11	Community-acquired infections	-	*bla*_KPC−2_
CAB19	ST11	Community-acquired infections	-	
CAB21	ST11	Community-acquired infections	*bla*_CTX−M_ group 1	*bla*_NDM−1_
CAU02	ST11	Community-acquired infections	*bla*_CTX−M_ group 1	-
ENV45	ST307	River water	*bla*_CTX−M_ group 1	-
ENV47	ST307	River water	*bla*_CTX−M_ group 1	-
CAU17	ST307	Community-acquired infections	*bla*_CTX−M_ group 1	-
ENV26	ST36	River water	-	-
CAU21	ST36	Community-acquired infections	-	-
ENV06	ST45	River water	*bla*_CTX−M_ group 1	-
ENV22	ST45	River water	-	-
CAB05	ST45	Community-acquired infections	-	-

*ESBL* extended-spectrum β-lactamase, *ST* sequence type

## Discussion

This study provides a comparative analysis of *K. pneumoniae* isolates from CA infections and an urban river, revealing overlapping antimicrobial resistance phenotypes and resistance determinants, but distinct patterns of virulence and population structure between the two settings. The high prevalence of resistance phenotypes observed in both sources, together with the detection of overlapping resistance determinants, highlights the occurrence of clinically relevant *K. pneumoniae* lineages beyond healthcare settings. Although higher resistance frequencies are typically reported among hospital-associated isolates (Asri et al. 2021), including in Salvador (Leal et al. 2019; Araujo et al. 2025), our findings indicate that clinically relevant resistance determinants are already established in non-clinical environments, representing a potential risk to human and animal health. Notably, the detection of ESBL- and carbapenemase-producing isolates in river water supports the role of the urban aquatic environment as a potential environmental reservoir (Grenni 2022), although the river may also reflect human contamination and reciprocal exchange of resistant *K. pneumoniae* between human community sources and environmental reservoirs.

Environmental detection of carbapenem-resistant *K. pneumoniae* has been reported previously in Brazil. In 2013, a river and lagoon survey conducted in Rio de Janeiro recovered seven carbapenem-resistant isolates (Araujo et al. 2016), whereas a more recent investigation in the same aquatic system did not identify carbapenem-resistant *Klebsiella* spp. (Martins et al. 2025). Such variation likely reflects the dynamic nature of environmental contamination, with detection influenced by local conditions, sampling strategy, and temporal fluctuations. Although the frequency of carbapenem-resistant isolates in our river samples was lower than that reported in other studies (Guo et al. 2023; Zou et al. 2023; Swain et al. 2025), their presence remains concerning, as even low-level environmental occurrence may contribute to the persistence and spread of clinically important pathogens. Importantly, the high fecal contamination observed in the river indicates a continuous input of human-associated bacteria, which may help account for the similarities in resistance profiles observed between environmental and CA isolates.

To further investigate whether similarities in phenotypic antimicrobial resistance were accompanied by shared resistance mechanisms, we compared the distribution of β-lactamase genes between CA infection isolates and river water isolates. The detection of ESBL- and carbapenemase-associated genes in isolates from both sources indicates that clinically relevant resistance determinants are present in both clinical and environmental settings. Importantly, the high frequency of *bla*_SHV_ detection should be interpreted cautiously, as it may largely reflect the intrinsic chromosomal *bla*_SHV_ gene commonly present in *K. pneumoniae*, rather than acquired ESBL-associated *bla*_SHV_ variants. Notably, all phenotypically ESBL-producing isolates carried genes belonging to the investigated CTX-M groups, consistent with the prominent role of CTX-M-type enzymes as ESBL determinants in *K. pneumoniae* across both clinical and environmental settings worldwide (Calbo and Garau 2015; Cho et al. 2023). Although studies specifically characterizing resistance determinants among CA *K. pneumoniae* isolates remain limited, CTX-M-group genes have also been frequently reported in environmental isolates. For example, a study from India detected *bla*_CTX−M_ in approximately 48% of *K. pneumoniae* isolates recovered from river water (Mondal et al. 2019).

Of particular relevance was the detection of *bla*_KPC−2_ and *bla*_NDM−1_ in isolates from both settings. Like *bla*_CTX−M_, these genes are frequently carried on mobile genetic elements, particularly conjugative plasmids, and environmental reservoirs may contribute to the persistence of resistance determinants in aquatic environments (Wyres and Holt 2018). Accordingly, the identification of carbapenemase-producing isolates in river water raises concern that urban aquatic environments may participate in the broader ecological circulation of carbapenem resistance. NDM-producing isolates are especially concerning, as metallo-β-lactamases are not inhibited by currently available β-lactamase inhibitors, leaving very limited therapeutic options (Mojica et al. 2022). KPC- and NDM-1–producing *K. pneumoniae* have been reported among hospital isolates in Salvador by our research group (Rocha et al. 2022; Silva et al. 2025), indicating that these resistance mechanisms are established in the local epidemiological scenario.

In contrast to antimicrobial resistance, hv*Kp* isolates were identified exclusively among clinical samples, and no convergence between antimicrobial resistance and hypervirulence was observed. The absence of acquired resistance determinants among hv*Kp* isolates is consistent with the classical description of hv*Kp* lineages, which have historically been characterized by broad antimicrobial susceptibility, although the emergence of MDR-hv*Kp* strains represents a growing global concern (Zhang et al. 2020). The restriction of hv*Kp* to clinical isolates in our collection may reflect the importance of hypervirulence-associated traits in host adaptation and infection, including immune evasion, enhanced iron acquisition, and tissue invasion. However, this finding should not be interpreted as evidence that hv*Kp* is absent from environmental reservoirs. Consistent with the greater representation of virulence-associated determinants among clinical isolates, yersiniabactin was significantly more frequent in clinical than in river water isolates (29.2% vs. 8.0%). Yersiniabactin contributes to iron acquisition and bacterial fitness during infection, supporting its potential relevance to host-associated *K. pneumoniae* populations (Bachman et al. 2011). Although hv*Kp* strains were not detected in river isolates in our study, they have been reported in environmental waters in East Asian settings (Zou et al. 2023; Liao et al. 2025). In these contexts, environmental detection may reflect the high prevalence of hv*Kp* in East Asian human populations and continuous human-associated shedding rather than specific adaptation to aquatic environments.

Compared with the clonal clustering often reported in hospital-based studies, particularly in outbreaks or among isolates from common sources, where high-risk clonal lineages often predominate (Wyres et al. 2020), the isolates in this study exhibited marked clonal heterogeneity, with 88 distinct STs identified among 115 strains. This finding indicates that the sampled population was not dominated by the expansion of a single introduced clone. Nevertheless, four STs were shared between river water and CA infection isolates, namely ST11, ST36, ST45, and ST307. Among them, ST11 and ST307 are recognized high-risk, internationally disseminated lineages (Wyres et al. 2020) and have been previously reported in hospital settings in Salvador (Rocha et al. 2022; Silva et al. 2025). The detection of ESBL- and carbapenemase-associated determinants among these lineages, particularly the carbapenemase genes *bla*_KPC−2_ and *bla*_NDM−1_ in ST11 isolates, further highlights their clinical relevance beyond healthcare settings. ST147, which was also identified in river water, has been described as an emerging antimicrobial-resistant high-risk lineage associated with global dissemination (Peirano et al. 2020). Although the presence of shared STs does not demonstrate direct transmission between environments, the occurrence of clinically relevant *K. pneumoniae* lineages in river water highlights the importance of urban aquatic environments in the broader epidemiology of antimicrobial resistance.

This study has limitations that should be considered when interpreting the findings. Environmental and clinical isolates were not geographically or temporally paired, which limits conclusions regarding direct transmission or epidemiological linkage between the two settings. In addition, although MLST allowed population comparison, its resolution is limited, and shared STs should not be interpreted as evidence of clonal relatedness. Whole-genome sequencing approaches would be required for higher-resolution comparisons. Although most environmental isolates (41/50) were recovered using a non-selective medium, nine isolates were obtained on cefotaxime-supplemented MacConkey agar. Therefore, the inclusion of isolates recovered under antimicrobial selection may have increased the representation of resistant phenotypes among environmental isolates and should be considered when interpreting comparisons of antimicrobial resistance frequencies between environmental and clinical isolates.

## Conclusions

This study demonstrates that *K. pneumoniae* isolates from community-acquired infections and from an urban river in Salvador, Brazil, comprise genetically diverse populations, with clinically relevant antimicrobial resistance determinants present in both settings, while virulence-associated characteristics differ between sources. Hypervirulent *K. pneumoniae* isolates were identified exclusively among clinical isolates, whereas high-risk lineages, including ST11 and ST307, were detected in both clinical and environmental settings and carried clinically relevant resistance determinants. Although the occurrence of shared STs across sources does not establish clonal relatedness or transmission, the detection of high-risk lineages and antimicrobial resistance determinants in river water highlights the potential role of non-healthcare environments as reservoirs of clinically relevant *K. pneumoniae*. These findings support a One Health perspective and reinforce the importance of integrating clinical, genomic, and environmental surveillance to better understand the distribution and potential circulation of high-risk *K. pneumoniae* lineages, as well as their resistance and virulence determinants.

## Supplementary Information

Below is the link to the electronic supplementary material.

Supplementary Material 1

## Data Availability

The dataset generated during and/or analysed during the current study is available in the Zenodo repository, https://doi.org/10.5281/zenodo.22735492.

## Declarations

### Daration of generative AI and AI-assisted technologies

ChatGPT was used solely for language editing, including grammar, spelling, punctuation, and wording.
