# Supplementary material for: Klebsiella pneumoniae from community-acquired infections and an urban river share antimicrobial resistance profiles and high-risk lineages but differ in virulence
Source: Arch Microbiol. 2026 Oct 6;208(12):681. doi: 10.1007/s00203-026-05239-0 (PMC13642446; doi:10.1007/s00203-026-05239-0)
Supplement: Supplementary file 1 [file 203_2026_5239_MOESM1_ESM.pdf]

Article title: *Klebsiella pneumoniae* from community-acquired infections and an urban river share antimicrobial resistance profiles and high-risk lineages but differ in virulence

Journal: Archives of Microbiology

Authors: Adriano Souza Santos Monteiro[1,2,†], João Ricardo Filho[1,3,†], Vívian Santos Galvão[4], Vanessa Tibolla Moretto[1], Ana Beatriz Purificação[1,5], Lee Senhorinha Almeida Andrade[1,5], Camila Maria Piñeiro Silva[4], Lúcio Macedo Barbosa[6], Luciano Kalabric Silva[1,5], Soraia Machado Cordeiro[4,7], Ronald Edward Blanton[8], Joice Neves Reis[4,5,7], Mitermayer Galvão Reis[1,3,5,9,10,*]

Corresponding Author: Mitermayer Galvão Reis

Affiliation: [1] Laboratory of Pathology and Molecular Biology, Gonçalo Moniz Institute, Oswaldo Cruz Foundation, Salvador, Bahia, Brazil; [2] Department of Biointeraction, Institute of Health Sciences, Federal University of Bahia, Salvador, Bahia, Brazil; [3] Postgraduate Program in Human Pathology, Gonçalo Moniz Institute, Oswaldo Cruz Foundation, Salvador, Bahia, Brazil; [4] Faculty of Pharmacy, Federal University of Bahia, Salvador, Bahia, Brazil; [5] Postgraduate Course in Biotechnology in Health and Investigative Medicine, Gonçalo Moniz Institute, Oswaldo Cruz Foundation, Salvador, Bahia, Brazil; [6] Bahiana School of Medicine and Public Health, Salvador, Bahia, Brazil; [7] Postgraduate Program in Microbiology, Institute of Biology, Federal University of Bahia, Salvador, Bahia, Brazil; [8] Department of Tropical Medicine, Tulane School of Public Health and Tropical Medicine, New Orleans, Louisiana, USA; [9] Faculty of Medicine, Federal University of Bahia, Salvador, Bahia, Brazil; [10] Yale School of Public Health, New Haven, Connecticut, USA

[†] A.S.S. Monteiro and J.R. Filho contributed equally to this work.

*Corresponding Author: Mitermayer Galvão Reis

E-mail: mitermayer.reis@fiocruz.br

Table S1. Primer sequences and PCR conditions for molecular characterization of *Klebsiella pneumoniae* isolates.

Target gene	Forward primer (5′–3′)	Reverse primer (5′–3′)	Amplicon size (bp)	Reference	PCR cycling conditions
bla$_{\text{TEM}}$	CATTTCCGTGTCGCCCTTATTC	CGTTCATCCATAGTTGCCTGAC	800	Dallenne et al. 2010	Initial denaturation: 94 °C, 4 min; Denaturation: 94 °C, 30 s; Annealing: 60 °C, 30 s; Extension: 72 °C, 1 min; Final extension: 72 °C, 7 min; 30 cycles
bla$_{\text{SHV}}$	AGCCGCTTGAGCAAATTAAAC	ATCCCGCAGATAAATCACCAC	713		
bla$_{\text{OXA-1-like}}$	GGCACCGATTCAACTTTCAAG	GACCCCAAGTTTCCTGTAAGTG	564		
bla$_{\text{CTX-M}}$ group 1	TTAGGAARTGTGCCGCTGYA	CGATATCGTTGGTGGTRCCAT	688		
bla$_{\text{CTX-M}}$ group 2	CGTTAACGGCACGATGAC	CGATATCGTTGGTGGTRCCAT	404		
bla$_{\text{CTX-M}}$ group 9	TCAAGCCTGCCGATCTGGT	TGATTCTCGCCGCTGAAG	561		
bla$_{\text{GES}}$	AGTCGGCTAGACCGGAAAG	TTTGTCCGTGCTCAGGAT	399		Initial denaturation: 94 °C, 4 min; Denaturation: 94 °C, 25 s; Annealing: 57 °C, 25 s; Extension:
bla$_{\text{OXA-48}}$	GCTTGATCGCCCTCGATT	TTCGGCTTGACTCGGCTGA	281		

Target gene	Forward primer (5′–3′)	Reverse primer (5′–3′)	Amplicon size (bp)	Reference	PCR cycling conditions
					72 °C, 30 s; Final extension: 72 °C, 7 min; 30 cycles
bla$_{\text{IMP}}$	TTGACACTCCATTTACDG	GATYGAGAATTAAGCCACYCT	139		Initial denaturation: 94 °C, 4 min; Denaturation: 94 °C, 25 s; Annealing: 55 °C, 25 s; Extension: 72 °C, 30 s; Final extension: 72 °C, 7 min; 30 cycles
bla$_{\text{VIM}}$	GATGGTGTTTGGTCGCATA	CGAATGCGCAGCACCAG	390		
bla$_{\text{KPC}}$	ATGTCACTGTATCGCCGTCT	TTTTCAGAGCCTTACTGCCC	893	Carattoli et al., 2021	Initial denaturation: 95 °C, 4 min; Denaturation: 95 °C, 30 s; Annealing: 58 °C, 30 s; Extension: 72 °C, 1 min; Final extension: 72 °C, 7 min; 30 cycles
bla$_{\text{NDM}}$	TGGCTTTTGAAACTGTCGCACC	CTGTCACATCGAAATCGCGCGA	1000	Gondal et al., 2020	Initial denaturation: 95 °C, 4 min; Denaturation: 95 °C, 30 s; Annealing: 61 °C, 30 s; Extension: 72 °C, 1 min; Final extension: 72 °C, 7 min; 30 cycles
ybtS	GACGGAAACAGCACGGTAAA	GAGCATAATAAGGCGAAAGA	242	Compain et al. 2014	Initial denaturation: 95 °C, 5 min; Denaturation: 94 °C, 30 s; Annealing: 60 °C, 60 s; Extension: 72 °C, 1 min; Final extension: 72 °C, 10 min; 30 cycles
mrkD	AAGCTATCGCTGTACTTCCGGCA	GGCGTTGGCGCTCAGATAGG	340		
entB	GTCAACTGGGCCTTTGAGCCGTC	TATGGGCGTAAACGCCGGTGAT	400		
K2	CAACCATGGTGGTCGATTAG	TGGTAGCCATATCCCTTTGG	531		
kfu	GGCCTTTGTCCAGAGCTACG	GGGTCTGGCGCAGAGTATGC	638		
allS	CATTACGCACCTTTGTCAGC	GAATGTGTCGGCGATCAGCTT	764		
magA	GGTGCTCTTTACATCATTGC	GCAATGGCCATTTGCGTTAG	1,283		
iroB	ATCTCATCATCTACCCTCCGCTC	GGTTCGCCGTCGTTTTCAA	235	Russo et al. 2018	Initial denaturation: 95 °C, 4 min; Denaturation: 95 °C, 30 s; Annealing: 59 °C, 30 s; Extension: 72 °C, 30 s; Final extension: 72 °C, 7 min; 25 cycles.
peg-344	AAAGGACAGAAAGCCAGTG	CAATGACGAGGGGGATAATC	411		Initial denaturation: 95 °C, 4 min; Denaturation: 95 °C, 30 s; Annealing: 53 °C, 30 s; Extension: 72 °C, 30 s; Final extension: 72 °C, 7 min; 25 cycles.
iucA	GCTTATTTCTCCCCAACCC	TCAGCCCTTTAGCGACAAG	583		Initial denaturation: 95 °C, 4 min; Denaturation: 95 °C, 30 s; Annealing: 59 °C, 30 s; Extension:

Target gene	Forward primer (5′–3′)	Reverse primer (5′–3′)	Amplicon size (bp)	Reference	PCR cycling conditions
					72 °C, 50 s; Final extension: 72 °C, 7 min; 25 cycles.
$_{p}$*rmpA*	GAGTAGTTAATAAATCAATAGCAAT	CAGTAGGCATTGCAGCA	332		Initial denaturation: 95 °C, 4 min; Denaturation: 95 °C, 30 s; Annealing: 50 °C, 30 s; Extension: 72 °C, 40 s; Final extension: 72 °C, 7 min; 25 cycles.
$_{p}$*rmpA2*	GTGCAATAAGGATGTTACATTA	GGATGCCCTCCTCCTG	430		
uge	GATCATCCGGTCTCCCTGTA	TCTTCACGCCTTCCTTCACT	534	Brisse et al. 2009	Initial denaturation: 94 °C, 5 min; Denaturation: 94 °C, 30 s; Annealing: 53 °C, 30 s; Extension: 72 °C, 50 s; Final extension: 72 °C, 7 min; 35 cycles.
wabG	CGGACTGGCAGATCCATATC	ACCATCGGCCATTTGATAGA	683		
ureA	GCTGACTTAAGAGAACGTTATG	GATCATGGCGCTACCT(C/T)A	337		Initial denaturation: 94 °C, 5 min; Denaturation: 94 °C, 30 s; Annealing: 55 °C, 30 s; Extension: 72 °C, 30 s; Final extension: 72 °C, 7 min; 35 cycles.
fimH	ATGAACGCCTGGTCCTTTGC	GCTGAACGCCTATCCCCTGC	688	El Fertas-Aissani et al. 2013	Initial denaturation: 94 °C, 3 min; Denaturation: 94 °C, 20 s; Annealing: 55 °C, 20 s; Extension: 72 °C, 43 s; Final extension: 72 °C, 7 min; 30 cycles.
traT	GGTGTGGTGCGATGAGCACAG	CACGGTTCAGCCATCCCTGAG	290		Initial denaturation: 94 °C, 4 min; Denaturation: 94 °C, 30 s; Annealing: 63 °C, 40 s; Extension: 72 °C, 1 min; Final extension: 72 °C, 7 min; 30 cycles.
hlyA	AACAAGGATAAGCACTGTTCTGGCT	ACCATATAAGCGGTCATTCCC GTCA	1177		
cnf-1	AAGATGGAGTTTCCTATGCAGGAG	CATTCAGAGTCCTGCCCTCATT ATT	498		Initial denaturation: 94 °C, 4 min; Denaturation: 94 °C, 30 s; Annealing: 56 °C, 40 s; Extension: 72 °C, 40 s; Final extension: 72 °C, 7 min; 30 cycles.
rpoB	GGCGAAATGGCWGAGAACCA	GAGTCTTCGAAGTTGTAACC	1075	Diancourt et al., 2005	Initial denaturation: 94 °C, 4 min; Denaturation: 94 °C, 20 s; Annealing: 50 °C, 30 s; Extension: 72 °C, 60 s; Final extension: 72 °C, 5 min; 35 cycles.
mdh	CCCAACTCGCTTCAGGTTCAG	CCGTTTTTCCCCAGCAGCAG	756		
pgi	GAGAAAAACCTGCCTGTACTGCTGG C	CGCGCCACGCTTTATAGCGGT TAAT	566		
phoE	ACCTACCGCAACACCGACTTCTTCG G	TGATCAGAACTGGTAGGTGAT	602		
infB	CTCGCTGCTGGACTATATTCG	CGCTTTCAGCTCAAGAACTTC	462		

Target gene	Forward primer (5′–3′)	Reverse primer (5′–3′)	Amplicon size (bp)	Reference	PCR cycling conditions
tonB	CTTTATACCTCGGTACATCAGGTT	ATTCGCCGGCTGRGCRGAGAG	539		Initial denaturation: 94 °C, 4 min; Denaturation: 94 °C, 20 s; Annealing: 45 °C, 30 s; Extension: 72 °C, 60 s; Final extension: 72 °C, 5 min; 35 cycles.
gapA	TGAAATATGACTCCACTCACGG	CTTCAGAAGCGGCTTTGATGGCTT	662		Initial denaturation: 94 °C, 4 min; Denaturation: 94 °C, 20 s; Annealing: 60 °C, 30 s; Extension: 72 °C, 60 s; Final extension: 72 °C, 5 min; 35 cycles.

Table S2. Phenotypic antimicrobial resistance profiles of *Klebsiella pneumoniae* isolates from community-acquired infections and river water.

**Phenotypic resistance profile**	**Community-acquired infections n = 65 (%)**	**River water n = 50 (%)**
No resistance	43 (66.2)	36 (72.0)
AMC, ATM, CAZ, CRO, FEP, CIP	4 (6.2)	2 (4.0)
AMC, TZP, ATM, CAZ, CRO, FEP, GEN, CIP	1 (1.5)	3 (6.0)
CIP	3 (4.6)	1 (2.0)
AMC, TZP, ATM, CAZ, CRO, FEP, IMP, MER, CIP	2 (3.1)	1 (2.0)
AMC, CIP	1 (1.5)	1 (2.0)
TZP	3 (4.6)	0 (0.0)
CAZ, CRO, FEP, GEN	2 (3.1)	0 (0.0)
GEN	1 (1.5)	0 (0.0)
AMC, TZP, CAZ, CRO, FEP, CIP	1 (1.5)	0 (0.0)
AMC, TZP, FEP, AK, GEN, CIP	1 (1.5)	0 (0.0)
ATM, CAZ, CRO, FEP, GEN, CIP	1 (1.5)	0 (0.0)
TZP, CAZ, CRO, FEP, IMP, MER, CIP	1 (1.5)	0 (0.0)
AMC, ATM, CAZ, CRO, FEP, GEN, CIP	1 (1.5)	0 (0.0)
AMC	0 (0.0)	1 (2.0)
ATM	0 (0.0)	1 (2.0)
AMC, CRO	0 (0.0)	1 (2.0)
ATM, CAZ, CRO	0 (0.0)	1 (2.0)
AMC, TZP, ATM, CAZ, CRO, FEP, CIP	0 (0.0)	1 (2.0)
AMC, TZP, ATM, CAZ, CRO, FEP, IMP, MER, GEN, CIP	0 (0.0)	1 (2.0)

AK, amikacin; AMC, amoxicillin-clavulanic acid; ATM, aztreonam; CAZ, ceftazidime; CIP, Ciprofloxacin; CRO, ceftriaxone; FEP, cefepime; GEN, gentamicin; IMP, imipenem; MEM, meropenem; TZP, piperacillin-tazobactam.

Table S3. Distribution of antimicrobial resistance among environmental isolates across river sampling sites.

**Antimicrobial agent**	**Sampling site***						
	**Site 1 (n = 1)**	**Site 2 (n = 2)**	**Site 3 (n = 2)**	**Site 4 (n = 1)**	**Site 5 (n = 14)**	**Site 6 (n = 10)**	**Site 7 (n = 20)**
Amoxicillin-clavulanic acid	-	-	-	1/1 (100%)	2/14 (14.3%)	2/10 (20.0%)	6/20 (30.0%)
Piperacillin-tazobactam	-	-	-	-	1/14 (7.1%)	-	5/20 (25.0%)
Ceftriaxone	-	-	-	-	2/14 (14.3%)	2/10 (20.0%)	6/20 (30.0%)
Ceftazidime	-	-	-	-	2/14 (14.3%)	2/10 (20.0%)	5/20 (25.0%)
Cefuroxime	1/1 (100%)	2/2 (100%)	1/2 (50%)	1/1 (100%)	14/14 (100%)	8/10 (80.0%)	20/20 (100%)
Cefepime	-	-	-	-	2/14 (14.3%)	1/10 (10.0%)	5/20 (25.0%)
Aztreonam	-	1/2 (50%)	-	-	1/14 (7.1%)	2/10 (20.0%)	5/20 (25.0%)
Ciprofloxacin	-	-	-	1/1 (100%)	2/14 (14.3%)	1/10 (10.0%)	6/20 (30.0%)
Gentamicin	-	-	-	-	-	-	4/20 (20.0%)
Imipenem	-	-	-	-	-	-	2/20 (10.0%)
Meropenem	-	-	-	-	-	-	2/20 (10.0%)
Multidrug-resistant	-	-	-	1/1 (100%)	2/14 (14.3%)	2/10 (20.0%)	6/20 (30.0%)

*Values are expressed as the number of resistant isolates over the total isolates tested at each site (n/N), with percentages in parentheses. "-" indicates tested isolates with no resistance detected.

Table S4. Distribution of β-lactamase genes detected in *Klebsiella pneumoniae* across sampling sites.

**Sampling site**	**β-lactamase genes**						**Total**
	***bla*TEM**	***bla*SHV**	***bla*OXA-1**	***bla*CTX-M**	***bla*KPC**	***bla*NDM**	
**Site 1**	-	1	-	-	-	-	1
**Site 2**	-	1	-	-	-	-	1
**Site 3**	-	-	-	-	-	-	0
**Site 4**	-	-	-	-	-	-	0
**Site 5**	1	11	-	2	-	-	14
**Site 6**	1	10	1	1	-	-	13
**Site 7**	7	17	5	3	1	1	34

Table S5. Novel sequence types, corresponding BIGSdb-Pasteur identifiers, and MLST allelic profiles.

Novel ST	Study ID	Source	BIGSdb Isolate	BIGSdb ID	*gapA*	*infB*	*mdh*	*pgi*	*phoE*	*rpoB*	*tonB*
ST8620	ENV37	River water	3K7.7	86866	2	1	66	1	122	4	13
ST8621	ENV39	River water	3K7.15	86867	17	19	11	39	9	21	52
ST8622	ENV14	River water	2K7.9	86865	18	22	18	16	11	13	1061
ST9648	ENV03	River water	1K2.4	144664	18	67	26	63	142	38	106
ST9649	ENV50	River water	4K7.22	144666	2	1	1	65	275	4	7
ST9650	ENV19	River water	3K5.4	144665	542	3	1	26	1	4	10
ST9651	CAB09	Community-acquired bloodstream infection	HB216	144668	3	1	720	1	1	1	4
ST9660	ENV10	River water	2K4.1	144678	38	19	53	628	73	21	130
ST9661	ENV16	River water	3K3.1	144679	16	24	227	27	55	22	188
ST9662	CAB07	Community-acquired bloodstream infection	HB185	144683	549	24	21	27	39	79	67
ST9663	ENV35	River water	3K7.5	144681	2	1	1	172	10	4	13
ST9664	CAU15	Community-acquired urinary tract infection	ITUAC440	144682	18	67	26	63	142	575	169
ST9758	CAU38	Community-acquired urinary tract infection	ITUAC473	144667	2	1	1	1	27	5	1411
ST9759	CAB17	Community-acquired bloodstream infection	HSR143	144669	38	19	53	40	73	21	1410

ST, sequence type.
